# Ageing-associated gut dysbiosis deteriorates mouse cognition

**DOI:** 10.3724/abbs.2024217

**Published:** 2025-02-13

**Authors:** Huihui Ju, Yile Zhou, Wanting Wei, Yan Hu, Hongwei Fang, Zhouyi Chen, Xia Sun, Yi Shi, Hao Fang

**Affiliations:** 1 Department of Anesthesiology Zhongshan Hospital Fudan University Shanghai 200032 China; 2 Department of Anesthesiology Minhang Branch Zhongshan Hospital Fudan University Shanghai 201199 China; 3 Department of Anesthesiology Shanghai Geriatric Medical Center Fudan University Shanghai 200032 China; 4 Shanghai Key Laboratory of Organ Transplantation Zhongshan Hospital Fudan University Shanghai 200032 China; 5 Department of Kidney Transplantation Zhongshan Hospital Fudan University Shanghai 200032 China

**Keywords:** ageing, cognition impairment, gut microbiota transplantation, *Lactobacillus*, acetic acid

## Abstract

Ageing is an independent factor for cognitive dysfunction. Ageing-associated alterations in the gut microbiota also affect cognition. The present study is designed to investigate changes in the gut microbiota and their participation in ageing-associated cognitive impairment. Both 10-week-old and 18-month-old mice are used. Mouse cognition is examined by novel object recognition and T-maze tests. Mouse feces are collected for sequencing and transplantation. Protein expression in the mouse intestine and hippocampus is studied using immunohistochemistry and immunofluorescence staining. Senescent neurons are induced by hydrogen peroxide *in vitro*. The cell lysates are used for western blot analysis and adenosine triphosphate (ATP) measurement. Our results show that 18-month-old mice exhibit cognitive dysfunction compared with young mice. In aged mice, transplanting the microbiota of young mice increases the protein presence of synaptophysin in the hippocampus and partially restores cognition. The protein expressions of mucin-2 and E-cadherin in the intestine are reduced in aged mice but are increased by transplantation. Gut microbiota analyses reveal that the reduced abundance of the microbe *Bacilli-Lactobacillales-Lactobacillaceae-Lactobacillus* in aged mice is restored by transplantation. Fecal microbiota transplantation in young mice increases the serum level of acetic acid in aged mice. Hydrogen peroxide stimulation induces senescence and reduces the protein expression levels of synaptophysin and acetyl-coenzyme A synthetase member 2 (ACSS2) in primary neurons. Incubation with acetic acid upregulates the protein expressions of ACSS2 and synaptophysin and further increases ATP production in senescent neurons. In summary, gut microbiota transplantation increases the abundance of *Lactobacillales*, elevates serum acetic acid level, and improves cognitive function in aged mice. Gut microbiota transplantation has therapeutic importance for ageing-associated cognitive decline.

## Introduction

Medical advances and hygiene improvements have significantly extended lifespan in modern society. However, the incidence of ageing-associated diseases, including neurological disorders, cardiovascular disease and malignant cancers, has also increased. It has been reported that more than 50 million senile people worldwide suffer from cognitive impairments, which will reach 130 million by 2050 [1]. Ageing-induced neuron impairment and microglial activation in the central nervous system are critical mechanisms underlying cognitive decline, shown as impaired learning capacity, memory loss, confusion, anxiety, and personality changes.

In addition to the well-studied mechanisms in the central nervous system, the gut microbiota plays a role in neurological disorders through the gut-brain axis [2], probably through the release of metabolites and neurotransmitters [3]. Notably, patients suffering from cognitive decline have altered gut microbiota communities, as shown by decreases in the beneficial bacteria *Akkermansia* and *Bifidobacterium* and increases in the detrimental bacteria *Ruminococcus* and *Eggerthella* [ 4– 7]. In a rodent model of Alzheimer’s disease, germ-free mice presented reduced cerebral amyloid-β plaques and blunted neurofibrillary tangles compared with their counterparts under specific pathogen-free conditions [8]. Transplanting the gut microbiota from Alzheimer’s disease model mice impaired memory in C57BL/6 mice [9]. Short-chain fatty acids (SCFAs), including acetic acid, pentanoic acid, hexanoic acid, isovaleric acid, and butyric acid, are the primary metabolites of bacteria. SCFAs are involved in regulating neurotransmitters [10], neurotrophic factors [11], myelination [12], and microglial homeostasis [13]. The administration of acetate increases the production of glutamate-glutamine, a regulatory neuropeptide, and suppresses appetite in mice [10]. Sodium butyrate increases mitochondrial biogenesis in astrocytes and ameliorates cognitive impairments in Alzheimer’s disease model mice [14]. Therefore, fecal microbiota transplantation (FMT) has been developed as an alternative treatment for cognitive impairment. Treatment with *Bacillus subtilis probiotics* inhibits α-synuclein aggregation in *C*. *elegans* [15]. In aged mice with cognitive dysfunction, probiotic treatment improved memory by restoring the anatomic structure of the gut and the blood-brain barrier [16].

Thus, the present study was designed to investigate changes in fecal microorganisms during aging and the underlying mechanisms of fecal microbiota transplantation in the cognition of aged mice.

## Materials and Methods

### Animals

Male C57BL/6 mice, 10 weeks and 18 months old, were purchased from Shanghai Jiesijie Company (Shanghai, China). The mice had free access to food and water. All animals were housed under specific pathogen-free conditions with 12/12-h light/dark cycles in the Laboratory Animal Unit of Zhongshan Hospital, Fudan University (Shanghai, China). After the cognition tests, the mice were euthanized with 1.2 g/kg urethane by intraperitoneal injection. Mouse serum, hippocampus, and intestine samples were collected. The experimental protocols were approved by the Animal Ethics Committee of Zhongshan Hospital, Fudan University (SYXK2021-#0022).

### Materials

Ampicillin, neomycin, metronidazole, and vancomycin were purchased from Macklin Biochemical (Shanghai, China). Papain was purchased from Aladdin (Shanghai, China). Neurobasal A medium, B27 supplement, glutamine, and the secondary antibodies Alexa Fluor488 and Alexa Fluor 594 were purchased from Thermo Fisher Scientific (Waltham, USA). DNase, acetic acid, and urethane were purchased from Sigma (St Louis, USA). Poly-D-lysine (ST-508), β-galactosidase kit (#C0602), and ATP assay kit were purchased from Beyotime (Shanghai, China). Antibodies against mucin-2 (ab272692), occludin (ab216327), zonula occludens-1 (ab221547), and protein-encoding gene product 9.5 (ab108986), neuronal nuclei antigen (NeuN, ab104224) and acyl-coenzyme A synthetase short-chain family member 2 (ACSS2, ab133664) were purchased from Abcam (Cambridge, UK). Antibodies against synaptophysin (17785-1-AP), lamin B (66095-1-Ig), microtubule-associated protein 2 (MAP2; 17490-1-AP), ACSS1 (17138-1-AP), ACSS3 (16204-1-AP), and β-actin (HRP-60008) were purchased from Proteintech (Wuhan, China). E-cadherin (3195S) and secondary antibodies against rabbit IgG were purchased from Cell Signaling Technology (Danvers, USA). Hydrogen peroxide was purchased from Sinopharm Chemical Reagent (Beijing, China).

### Fecal microbiota preparation and transplantation

Fresh fecal pellets from young and aged mice were collected and pooled. Fecal samples were prepared with sterile phosphate-buffered saline (PBS; 150 mg/mL). After being steeped for 15 min, the fecal supernatant was aliquoted and stored at -80°C.

**Figure 1:**
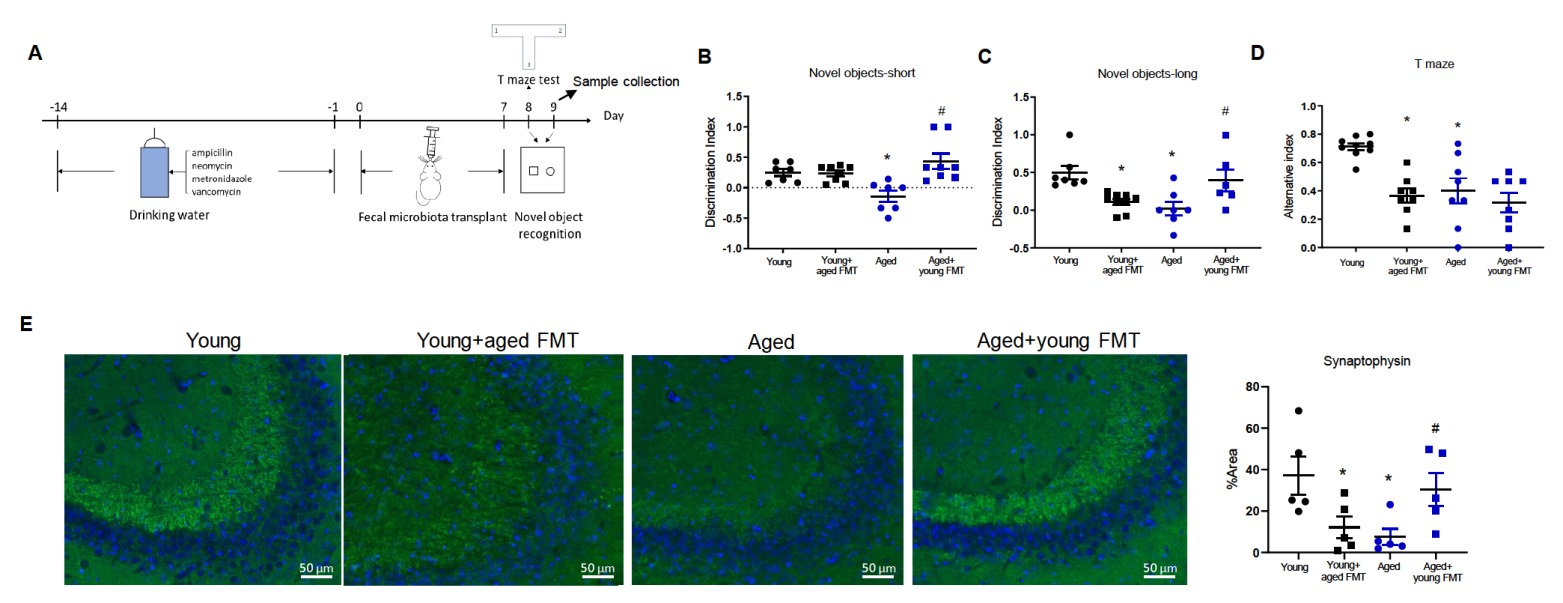
Figure 1 Fecal microbiota transplantation improves cognitive function in aged mice (A) Working diagram of the experiments. (B) Short-term memory. (C) Long-term memory. (D) Spatial orientation tests. n = 7–11. *P < 0.05. (E) Immunofluorescence signals of synaptophysin in the CA3 area of the mouse hippocampus. n = 5. Blue represents DAPI, green represents synaptophysin. Magnification, 200×. n = 6–8. *P < 0.05 compared with young control mice; #P < 0.05 compared with aged mice.

Before FMT, broad-spectrum antibiotics, including ampicillin (1 g/L; A800200), neomycin (1 g/L; 814740), metronidazole (1 g/L; M813526), and vancomycin (0.5 g/L; V871983), were dissolved in the drinking water for two weeks. The bacterial suspension (100 μL) and solvent control PBS were administered to recipient mice by gavage for seven consecutive days ( Figure 1A) [ 17, 18].

### Novel object recognition test

The novel object recognition test was used to examine mouse memory [19]. The mice were accustomed to two identical objects for 10 min in the chamber. In the experiment, one of the two objects was replaced by a novel subject. Mouse short-term memory was examined within 1 h, and long-term memory was examined within 24 h. Mouse movement was recorded during the experiments.

### Spontaneous alternation in the T-maze

Spontaneous alternation was examined in a T-maze [19]. A successful entry was counted when a mouse entered an arm of the maze. The mice were credited when they entered the three different arms in three consecutive entries, not credited when they discontinuously chose one arm, and credited minus when they repeatedly entered the same arm in three consecutive entries. The final scores were calculated for 5 min or a total of 15 entries.

### Gut microbiota analysis

Mouse stool was examined via 16S rRNA sequencing (Oebiotech, Shanghai, China). Bacterial diversity was analyzed by amplifying the V3-V4 variable regions. The sequencing data were normalized by noise elimination, fragment combination, and base correction. The valid tags were clustered into operational taxonomic units [20]. The gut microbiome composition was analyzed at the phylum, class, order, family, and genus levels. The diversity of the microbiota was examined using the Shannon diversity index. Bioinformatics analysis was performed using OECloud tools ( https://cloud.oebiotech.com).

### Fecal 16S rDNA assessment

The feces were extracted with a Stool DNA Kit (18820ES; Yeasen, Shanghai, China). The extracted total fecal bacterial DNA was quantified using a NanoDrop 1000 spectrophotometer (Thermo Fisher Scientific). Quantitative polymerase chain reaction was carried out with SYBR Green Master Mix (11200ES08; Yeasen). The primer sequences for 16S are as follows: F: 5′-TATTACCGCGGCTGCTGG-3′, and R: 5′-AGACACGGTCCAGACTCCTACG-3′.

### Short-chain fatty acid assessment

Serum levels of short-chain fatty acids were measured by liquid chromatography with tandem mass spectrometry (Oebiotech). Briefly, serum samples (100 μL) were mixed with 100 μL of 50% acetonitrile pre-chilled at 4°C and centrifuged at 12,000 *g* for 10 min. For the derivatization of SCFAs, 80 μL of supernatant was mixed with 40 μL of 200 mM 3-nitrophenylhydrazine and 40 μL of 120 mM N-(3-dimethylaminoprony)-N′-ethylcarbodiimede hydrochloride for 30 min at 40°C. The LC-MS/MS analysis of sample was conducted on QTRAP 5500+ mass spectrometer (AB Sciex, Harrogate, UK). The raw data were collected and processed using AB Sciex quantitative software. The mass spectral peak area of the sample analyte was substituted into a linear equation to calculate the concentration.

### Hematoxylin and eosin (H&E) staining, immunohistochemical staining and immunofluorescence staining

After euthanization, the mice were perfused with 20 mL of ice-cold PBS and 20 mL of 4% paraformaldehyde. The brains were fixed in 4% paraformaldehyde for 4 h, dehydrated in 30% (w/v) sucrose solution for three days, and embedded in optimum cutting temperature compound (Sakura, San Diego, USA) for immunofluorescence staining. The intestine samples were cut to 5 μm thickness, and the brain tissue was prepared at 40 μm thickness. The slices were stained with hematoxylin-eosin, then moved into 80%, 95%, 100% anhydrous alcohol for comcompletely dehydrated for H&E staining. For immunohistochemical and immunofluorescence staining, the slices were blocked with 5% goat serum and then incubated with primary antibodies (1:100) at 4°C overnight. On the second day, the slices were incubated with secondary antibodies (1:500) for 1 h. 3,3’-diaminobenzidine was used as the chromogen in immunohistochemistry. Images were captured via a fluorescence microscope (BX51; Olympus, Tokyo, Japan). The quantification of protein expression was conducted via ImageJ (NIH, Bethesda, USA).

### Primary hippocampal neuron culture

Primary hippocampal neuron culture was performed as previously reported [19]. Briefly, neurons were isolated from newborn hippocampi with papain (#P164463) and DNase (#DN25). After being filtered through a 40-μm strainer, 1 × 10 ^6^ neurons were seeded in poly-D-lysine (#ST-508)-coated 3.5-cm dishes and cultured with neurobasal A medium (#10888022) supplemented with B27 (#17504) and glutamine (0.5 mM; #35050). The medium was replaced on the second day (2 mL), and fresh neurobasal A medium (200 μL) was added to the dishes on day 7. On day 9, the neurons were challenged with hydrogen peroxide [ 21, 22]. Acetic acid (1 μM), according to the SCFA measurement in serum, was added to the culture medium 24 h before hydrogen peroxide (#695092) stimulation.

### Western blot analysis

Total protein from cultured cells was prepared with lysis buffer (150 mM NaCl, 1 mM EDTA, 1 mM NaF, 1 mM dithiothreitol, 10 μg/μL aprotinin, 10 μg/μL leupeptin, 0.1 mM Na _3_VO _4_, 1 mM phenylmethylsulfonyl fluoride, and 0.5% NP-40). Protein extracts (20 μg) were separated by 10%–12.5% sodium dodecyl sulfate-polyacrylamide gel electrophoresis, and then transferred to polyvinylidene fluoride membranes (0.45 μm; Merck Millipore, Darmstadt, Germany). After being blocked with 5% nonfat milk for 1 h, the membranes were incubated with primary antibodies (1:1000) at 4°C overnight. On the second day, the membranes were incubated with secondary antibodies against rabbit IgG (1:1000) for 1 h. Then, membranes were washed and the bands were visualized using an ECL kit (Epizyme, Shanghai, China). Protein expression was normalized to that of β-actin via ImageJ.

### Beta-galactosidase measurement

Cell senescence was evaluated by measuring β-galactosidase signals as reported previously [23]. Cultured neurons were fixed with paraformaldehyde and incubated with β-galactosidase staining buffers and X-Gal at 37°C overnight.

### Adenosine triphosphate (ATP) assessment

The ATP concentration was assessed with an ATP assay kit (#S0026) and detected with a luminometer (Thermo Fisher Scientific). In brief, cultured cells were collected with cell lysate and centrifuged at 12,000 *g* for 5 min at 4°C. The supernatant was collected for subsequent ATP assays.

### Cell cytotoxicity assay

Cell cytotoxicity was evaluated on the basis of LDH release into the extracellular medium using LDH cytotoxicity assay kit (C0016; Beyotime) according to the manufacturer’s instructions. In brief, cultured cells were incubated with LDH release reagent for 1 h. Cultured medium were collected and incubate with LDH working solution for 30 min at room temperature in the dark. The absorbance was measured at 490 nm.

### Statistical analysis

GraphPad Prism 9 (GraphPad Software, San Diego, USA) was used in the present study. Data are presented as the mean ± SEM. The statistical analysis was performed using one-way ANOVA followed by a *post hoc* Bonferroni comparison. The conjoint analysis of SCFAs and the microbiota was conducted via OECloud tools. The correlations between SCFAs and the microbiota were determined via Spearman correlation. Statistical difference was considered significant when the *P* value was less than 0.05.

## Results

### Fecal microbiota transplantation alters age-related cognition in mice

Mouse memory and spatial orientation were examined using the novel object recognition and T-maze tests, respectively [19]. Compared with young mice, aged mice had lower performance scores in short-term and long-term memory tests ( Figure 1B,C). In the T-maze test, aged mice also performed worse than their young counterparts did ( Figure 1D).

Aged mice transplanted with young fecal microbiota had improved scores in short-term and long-term memory tests but not in spatial orientation tests. Young mice transplanted with the fecal microbiota from aged mice had lower scores in the long-term memory and spatial orientation tests but not in the short-term memory test ( Figure 1B–D).

Immunofluorescence staining revealed that the protein expression of synaptophysin, a presynaptic protein in neurons, was lower in the hippocampus of aged mice than in that of young mice. The protein expression of synaptophysin was reduced in young mice transplanted with fecal microbiota from aged mice and increased in aged mice transplanted with young fecal microbiota ( Figure 1E).

### FMT alters the intestinal microenvironment in aged mice

**Figure 2:**
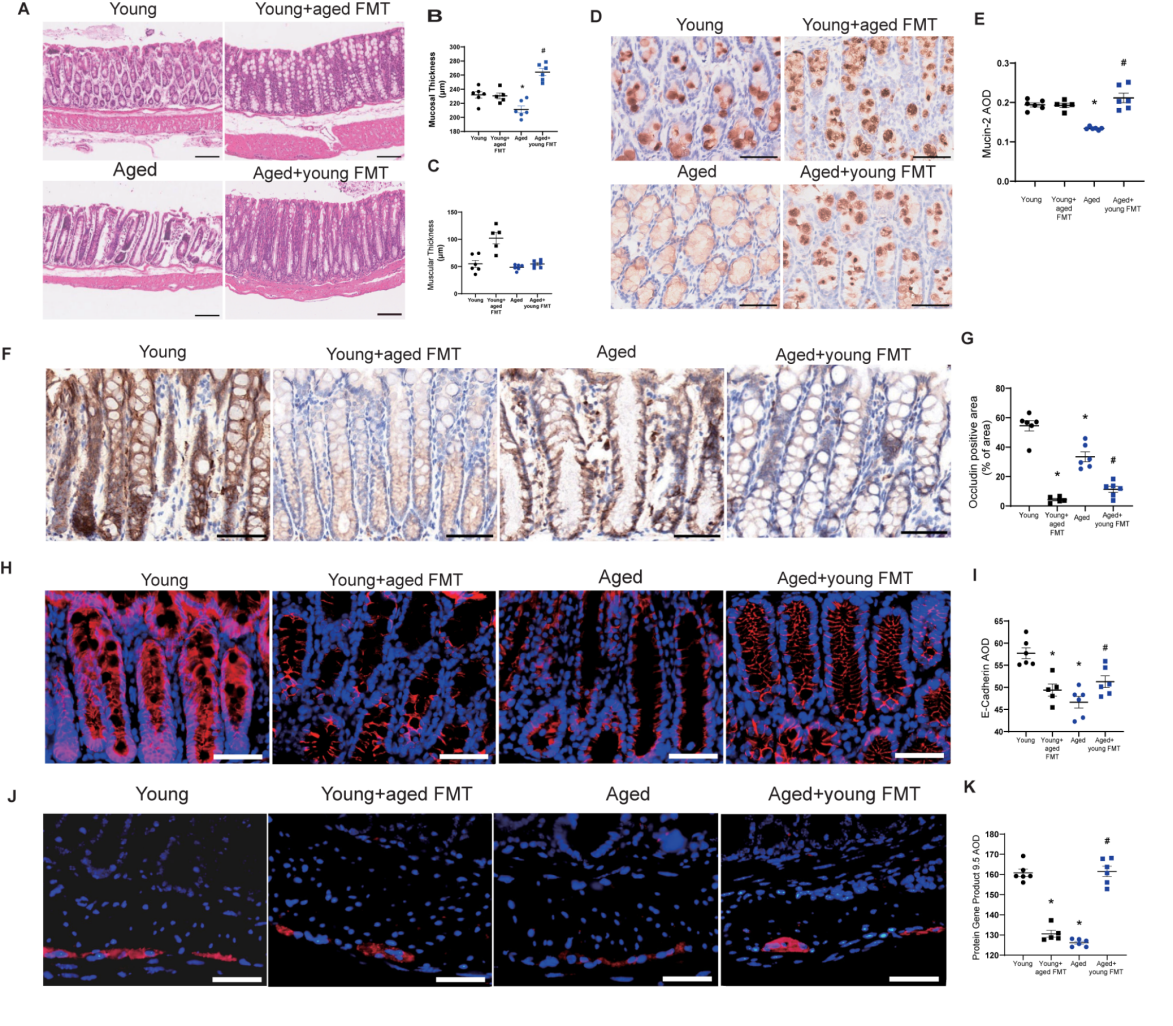
Figure 2 Fecal microbiota transplantation alters the intestinal microenvironment in aged mice (A) H&E staining of the colon intestine. Magnification, 100×. (B,C) Quantitative analysis of mucosal thickness (B) and muscular thickness (C). (D–G) Immunohistochemical staining (D,F) and quantification (E,G) of Mucin-2 (D,E) and occludin (F,G) in the mouse intestine. (H–M) Images of immunofluorescence staining (H,J,L) and quantification of the immunofluorescence intensity (I,K,M) of ZO-1, E-cadherin, and PGP9.5. Magnification, 500×. n = 5–6. *P < 0.05 compared with young control mice; #P < 0.05 compared with aged mice.

The gastrointestinal microenvironment is composed of intestinal parenchymal cells, the enteric immune system, and the enteric nervous system. H&E staining revealed that aged mice presented a thinner mucosal layer and disarrangement of the intestinal glands ( Figure 2A–C). The protein level of mucin-2, a secreted glycoprotein separating the gut microbiome from the mucosa [ 24, 25], was lower in aged mice than in young mice ( Figure 2D,E). In addition, aged mice presented reduced levels of occludin, ZO-1, and E-cadherin. FMT with the fecal microbiota from young mice restored the mucosal layer and significantly increased the protein expressions of mucin-2 and E-cadherin, but not occludin or ZO-1, in aged mice. FMT from the fecal microbiota of aged mice did not affect the mucosal layers or protein expressions of mucin-2 and ZO-1 in young mice ( Figure 2F–K). Compared with the control, FMT significantly reduced the protein level of occludin in both young and aged mice ( Figure 2F,G).

The protein expression of protein-encoding gene product 9.5 (PGP9.5), a marker of intestinal neurons [ 26, 27], was lower in aged mice than in young mice. FMT to the fecal microbiota of young mice significantly increased the expression of PGP9.5 in aged mice. FMT with the fecal microbiota from aged mice significantly reduced the expression of PGP9.5 in young mice ( Figure 2L,M).

### FMT alters the gut microbiota in aged mice

**Figure 3:**
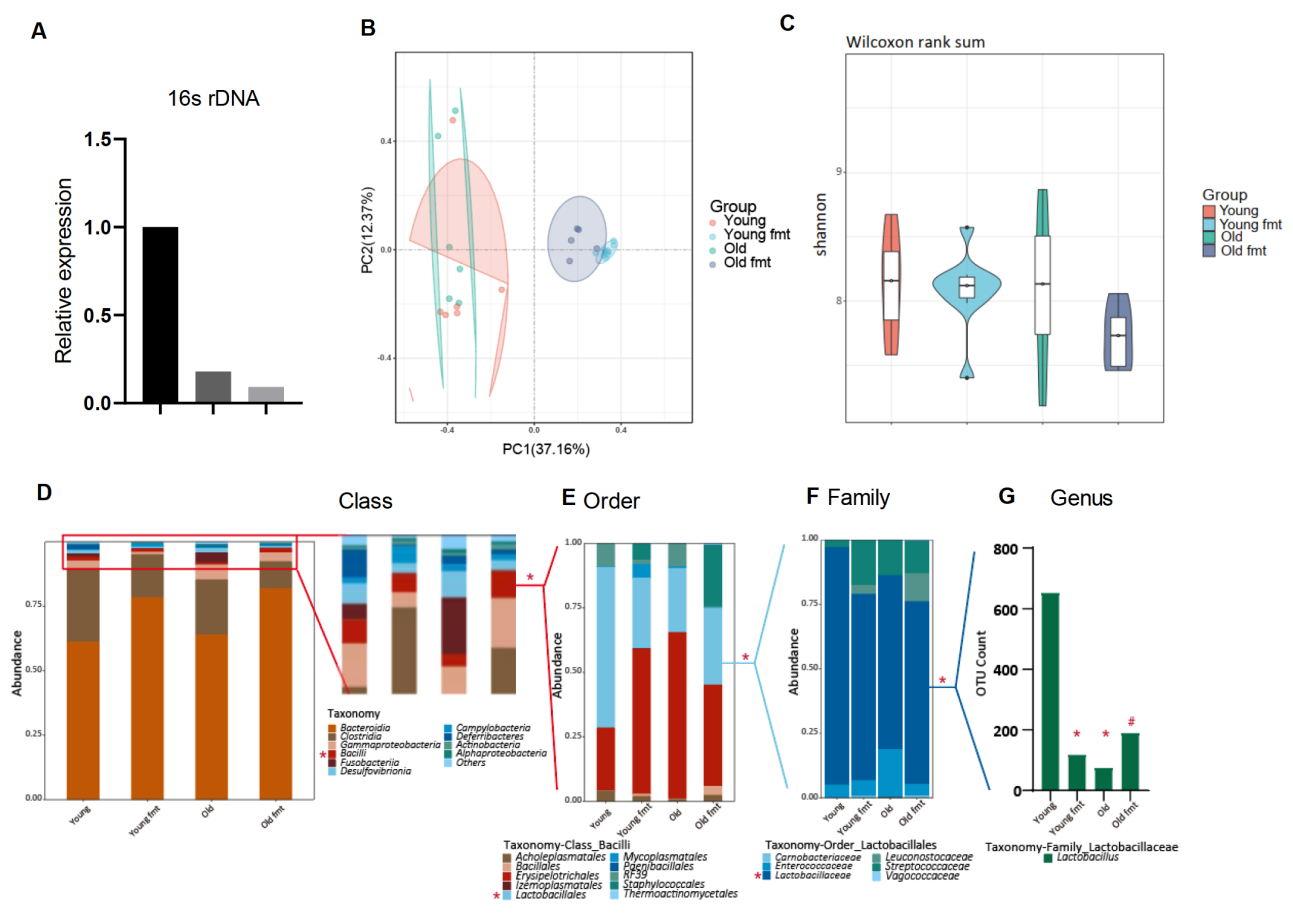
Figure 3 Fecal microbiota transplantation improves gut microbiota homeostasis in aged mice (A) Verification of the elimination of the gut microbiota by antibiotics. (B) PCoA of the gut microbiota. (C) Shannon index of the gut microbiota. (D–G) Community structure of the gut microbiota according to class (D), order (E), family (F), and genus rank (G). *P < 0.05 compared with young control mice, #P < 0.05 compared with aged mice.

To study the dysbiosis of gut bacterial communities in aged mice, the gut microbiota and serum short-chain fatty acids were measured and sequenced. Antibiotic administration eliminated 90% of the fecal microbiota ( Figure 3A). Principal coordinate analysis revealed that the stool of the mice that underwent FMT was less variable than that of the control mice, since the mouse fecal samples were pooled for transplantation ( Figure 3B). The Shannon analysis did not detect significant differences in the biodiversity of the fecal microbiota between the groups ( Figure 3C). The community structure revealed that the class *Bacteroidia* was dominant in the gut microbiota, followed by *Clostridiales*, *Gammaproleodactaris*, *Bacilli*, *Fusobacterlia*, *Desulfovibrionia*, *Campylobacteria*, *Deferribacteres*, *Actinobacteria*, and *Alphaproteobacteria*. The abundance of *Bacilli*, but not other microbes, was significantly altered in aged mice. *Bacilli* and their lower-ranked microorganisms, *Bacilli-Lactobacillales-Lactobacillaceae-Lactobacillus* (class-order-family-genus), were significantly reduced in aged mice. FMT with feces from young mice restored the abundance of *Bacilli-Lactobacillales-Lactobacillaceae-Lactobacillus* in aged mice, and FMT with the fecal microbiota from aged mice resulted in a reduced abundance of *Bacilli-Lactobacillales-Lactobacillaceae-Lactobacillus* in young mice ( Figure 3D‒G and Supplementary Figures S1–4).

**Figure 4:**
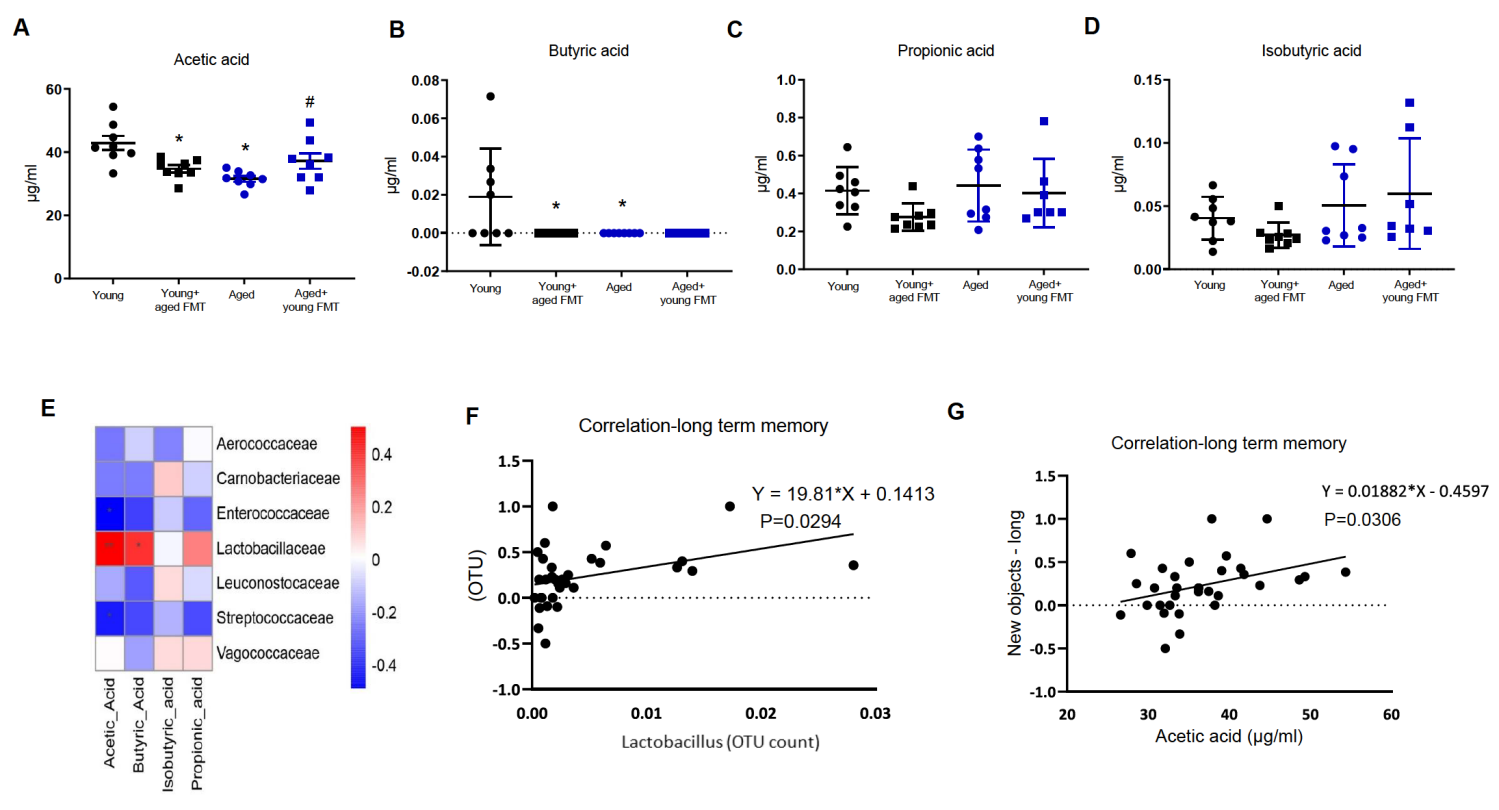
Figure 4 Fecal microbiota transplantation alters the serum levels of short-chain fatty acids (A–D) Serum concentrations of acetic acid (A), butyric acid (B), propionic acid (C), and isobutyric acid (D). (E) Correlation analysis of short-chain fatty acids and the gut microbiota of Lactobacillus. Red indicates a positive correlation, blue indicates a negative correlation. *P < 0.05. (F) Correlation analysis of the abundance of Lactobacillus and long-term memory. (G) Correlation analysis of the serum level of acetic acid and long-term memory. *P < 0.05 compared with young control mice; #P < 0.05 compared with aged mice. n = 8.

SCFAs, which are simple carboxylic acids with one to six carbon atoms, are the primary metabolites of the gut microbiota. The serum levels of acetic acid and butyric acid, but not propionic acid or isobutyric acid, were lower in aged mice than in young mice. FMT with feces from aged mice significantly reduced acetic acid and butyric acid levels in young mice. FMT with feces from young mice increased the level of serum acetic acid ( Figure 4A–D).

The abundance of *Lactobacillaceae* was positively correlated with the levels of acetic acid and butyric acid, whereas the abundances of *Enterococcaceae* and *Streptococcaceae* were negatively correlated with the plasma levels of acetic acid ( Figure 4E). The abundance of *Lactobacillus* and the serum level of acetic acid were significantly correlated with mouse performance scores during long-term memory examination ( Figure 4F,G and Supplementary Figure S5).

### Acetic acid modulates synaptic protein expression in cultured neurons

FMT significantly increased the protein presence of synaptophysin in the hippocampus and PGP9.5 in the intestines of aged mice, suggesting that FMT has protective effects on neurons. We isolated and identified mouse primary neurons from neonatal mouse ( Supplementary Figure S6). To mimic aging, primary hippocampal neurons were induced with hydrogen peroxide [ 21, 22]. Acetic acid was added to the culture medium, since acetic acid is taken up by the brain [28].

**Figure 5:**
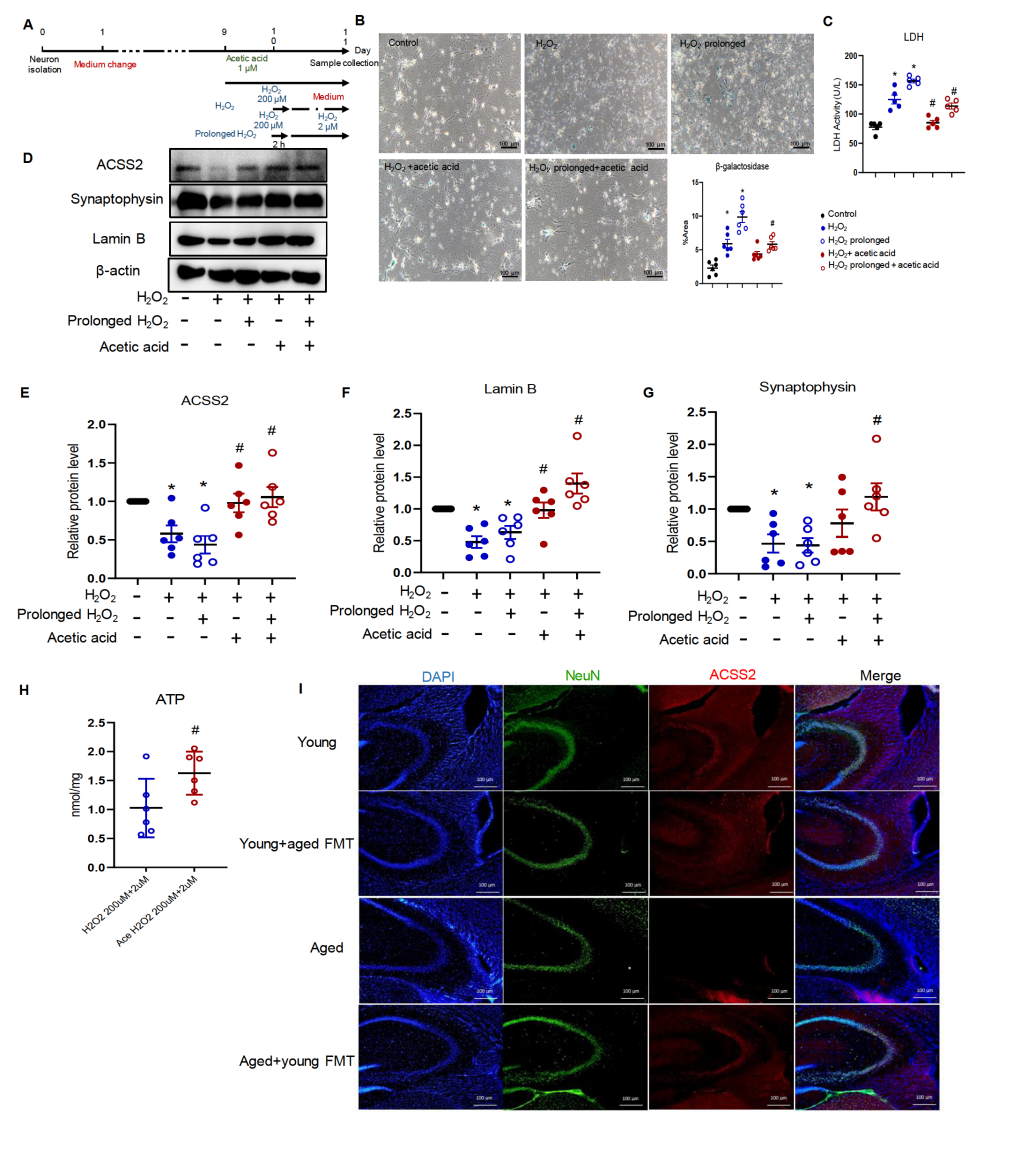
Figure 5 Acetic acid increases synaptic protein expression in neurons (A) Working diagram of the cell culture experiments. (B) β-Galactosidase staining of cultured primary neurons. Magnification, 100×. (C) LDH measurement. (D–G) Representative of western blots (D) and quantification of ACSS2 (E), lamin B (F), synaptophysin (G), and β-actin. (H) ATP levels in senescent neurons subjected to prolonged stimulation with hydrogen peroxide. n = 7. (I) Immunofluorescence signals of ACSS2 in the CA3 area of the mouse hippocampus. Blue represents DAPI, green represents NeuN, and red represents ACSS2. Magnification, 100×. *P < 0.05 compared with cells under the control condition; #P < 0.05 compared with cells with prolonged hydrogen peroxide incubation. n = 3.

The results showed that both temporal and prolonged exposure to hydrogen peroxide significantly increased β-galactosidase signals and downregulated lamin B protein expression in cultured neurons ( Figure 5B,D,F). Hydrogen peroxide significantly increased LDH level in cultured medium ( Figure 5C). The protein expression of synaptophysin was downregulated in neurons challenged with prolonged incubation with hydrogen peroxide. Incubation with acetic acid restored synaptophysin protein expression in senescent neurons and significantly reduced LDH level ( Figure 5C,D,G).

Acetyl-coenzyme A synthetase (ACSS) converts acetic acid to acetyl-CoA, the crucial player in citric acid cycle and ATP production [29]. Prolonged stimulation reduced the protein expression of ACSS2 in senescent neurons, which was significantly increased by incubation with acetic acid ( Figure 5D,E). Acetic acid treatment of cultured neurons subjected to prolonged hydrogen oxide stimulation significantly increased ATP production ( Figure 5H).

Consistently, FMT increased the protein expression of ACSS2, but not ACSS1 or ACSS3, in the hippocampal neurons of aged mice ( Figure 5I and Supplementary Figure S7).

## Discussion

The present study revealed that neuron dysfunction, as well as neuron loss, is a critical mechanism underlying aging and aging-associated cognitive impairment [ 30, 31]. Ageing reduces the abundance of *Bacilli-Lactobacillales-Lactobacillaceae-Lactobacillus* and decreases the serum level of acetic acid. FMT treatment increases serum level of acetic acid, mitigates neuron dysfunction, and improves cognition in aged mice.

Ageing is an inevitable biological process but presents some pathological characteristics resembling those of disease conditions. The mechanisms underlying ageing-associated cognitive impairment include neuronal death [19], synaptic abnormalities [9], microglial activation [ 32, 33], and disruption of the blood-brain barrier [ 34, 35]. In the present study, the reduced presence of synaptophysin, a synaptic protein, in aged mice suggests that neuron dysfunction and synaptic loss occur in ageing-associated cognitive impairment [ 30, 31].

The participation of the gut-brain axis under physiological and pathological conditions has been studied. On the one hand, the brain regulates gut movement, intestinal secretion, and sensory functions; on the other hand, the gut microbiota also releases signals affecting brain function [3]. It has been reported that treatment with *Bacillus* alleviates Parkinson’s disease by protecting against α-synuclein aggregation in an established *Caenorhabditis elegans* model of synucleinopathy [15]. Treatment with *Lactobacillus* reversed social deficits in a mouse model of autism spectrum disorder [36]. Commercial products of *Lactobacillus* improve cognition by protecting the intestinal and blood‒brain barriers in aged mice [16] and restoring learning and memory function in a vascular dementia model in rats [37]. In the present study, FMT with young feces partially restored the intestinal barrier and significantly improved cognition in aged mice, suggesting that the gut microbiota transplantation is a promising strategy for treating neurological disorders [ 38– 41].

Importantly, the abundance of the microbe *Bacilli* and its lower-ranked microorganisms is significantly reduced in aged mice and restored in aged mice treated with young feces, confirming the crucial involvement of *Bacilli-Lactobacillales-Lactobacillaceae-Lactobacillus* in aging-associated cognitive impairment [42]. *Lactobacillus* is a critical microorganism that produces organic acid, including lactic acid and acetic acid [ 43– 45]. Similarly, fecal SCFA levels were significantly higher in a centenarian population than in their younger counterparts in a Chinese regional study [46]. Impaired acetic acid metabolism has been reported in rodents subjected to hepatic ischaemia-reperfusion injury [47], chronic cerebral hypoperfusion [48], spontaneous hypertension [49], Coffin-Siris syndrome [50], and sleep disruption-induced cognitive impairment [51]. The underlying mechanisms include downregulating inflammation and oxidative stress in microglia in the mouse hippocampus by binding to GPR43 [52], restoring N-methyl D-aspartate receptor-induced histone hypoacetylation [53], improving neural development and synaptic transmission by increasing H3K27 acetylation in excitatory neurons [50], promoting neuronal cell growth through activation of the BDNF/TrkB signaling pathway in neuronal cells [54], and restoring glycolysis in astrocytes [51]. Indeed, the abundance of *Lactobacillus* is positively related to acetic acid levels and cognitive scores in aged mice. The increase in synaptophysin expression in cultured neurons caused by acetic acid further confirms the protective effects of acetic acid on neurons.

In the present study, hydrogen peroxide increased β-galactosidase signals and reduced lamin B expression in cultured cells, confirming that increased oxidative stress is critical in ageing [23]. Notably, prolonged, but not short, exposure to hydrogen peroxide significantly reduces the protein expression of synaptophysin, indicating that functional changes precede phenotypic changes.

The ACSS2 protein converts acetate into acetyl-CoA for histone acetylation [ 29, 47, 55]. ACSS2 has been reported to regulate dorsal hippocampus-mediated long-term spatial memory via the upregulation of neuronal plasticity-related immediate-early genes [56]. Consistently, acetic acid upregulates ACSS2 protein expression and increases ATP production, leading to increased synaptophysin expression in senescent neurons, suggesting that *Lactobacillus*-regulated acetic acid protects neurons by regulating citric acid cycle and ATP production [ 57– 59].

Furthermore, the pathological changes in the intestinal wall and reduced protein presence of junction proteins in gastrointestinal epithelial cells in aged mice confirmed aging-induced gastrointestinal changes. FMT treatment in young mice restored E-cadherin expression, suggesting that FMT, at least partially, improved the gastrointestinal barrier. Nevertheless, the presence of occludin is reduced by the FMT procedure in both young and aged mice, suggesting that FMT affects cell junctions. The upregulation of E-cadherin in aged mice subjected to FMT from young feces deserves further investigation.

In conclusion, the present study revealed that the beneficial microbe *Lactobacillus* and its primary metabolite acetic acid increase ATP production in cultured neurons and improve cognition in aged mice. Therefore, microbiota transplantation or corresponding treatments regulating the gut microbiota constitute an alternative or auxiliary approach for people with cognitive impairments.

## Supporting information

24506Supplementary_figures

## Supplementary Data

Supplementary data is available at *Acta Biochimica et Biophysica Sinica* online.
