# Supplementary material for: Ageing-associated gut dysbiosis deteriorates mouse cognition: Ageing-associated gut dysbiosis deteriorates mouse cognition
Source: Acta Biochim Biophys Sin (Shanghai). 2025 Feb 13;57(8):1234–43. doi: 10.3724/abbs.2024217 (PMC12368526; doi:10.3724/abbs.2024217)
Supplement: 24506Supplementary_figures [file 24506Supplementary_figures.pdf]

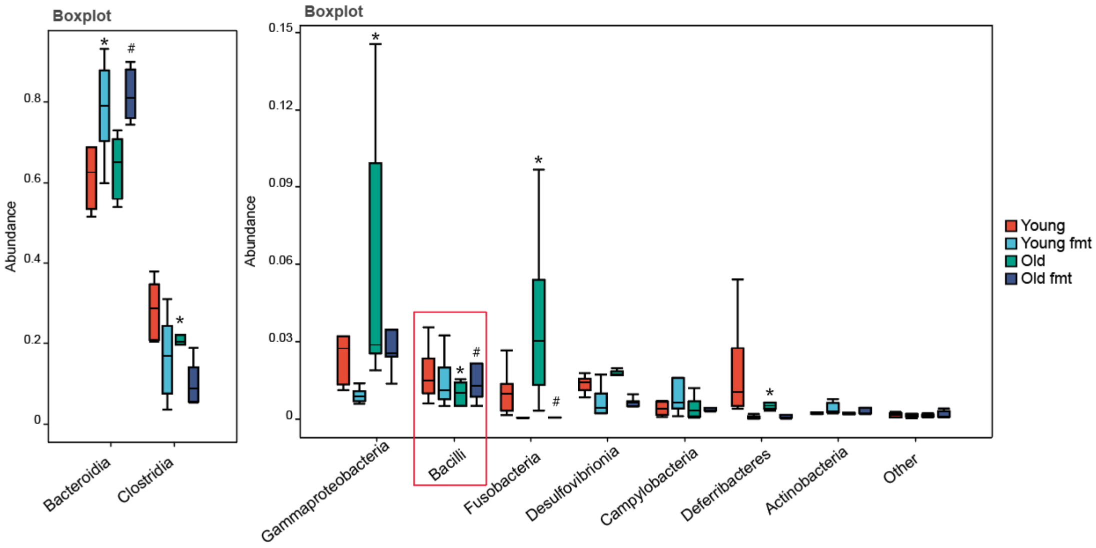

Supplementary Figure S1. Abundance of gut microbiota in class rank *$P<0.05$ compared with young control mice, #$P<0.05$ compared with aged mice.

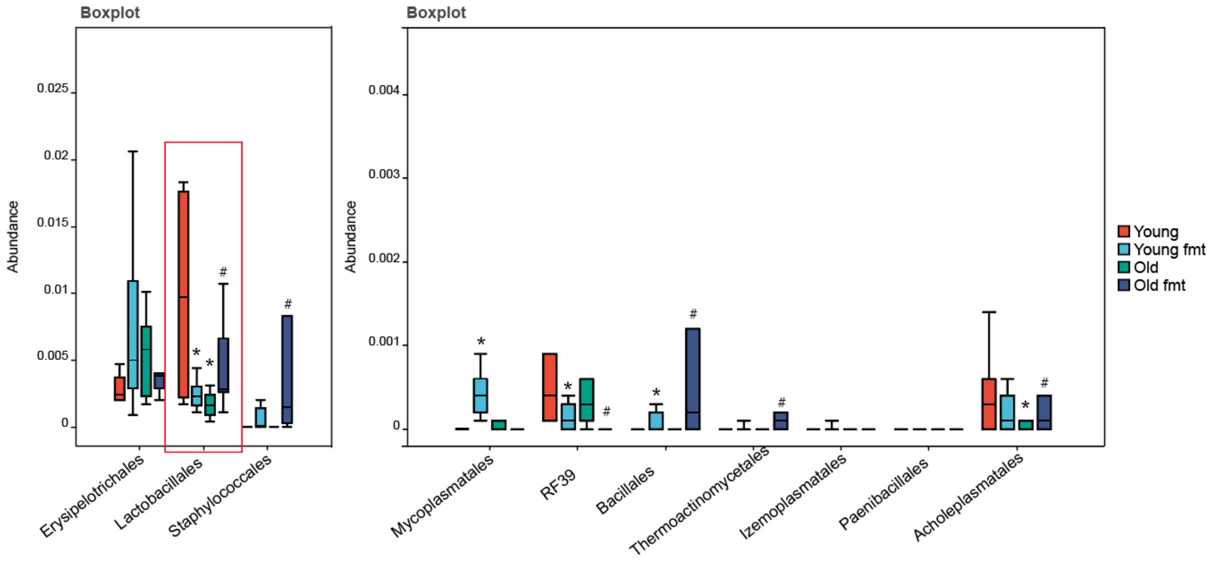

Supplementary Figure S2. Abundance of gut microbiota in *Bacilli* **P*<0.05 compared with young control mice, #*P*<0.05 compared with aged mice.

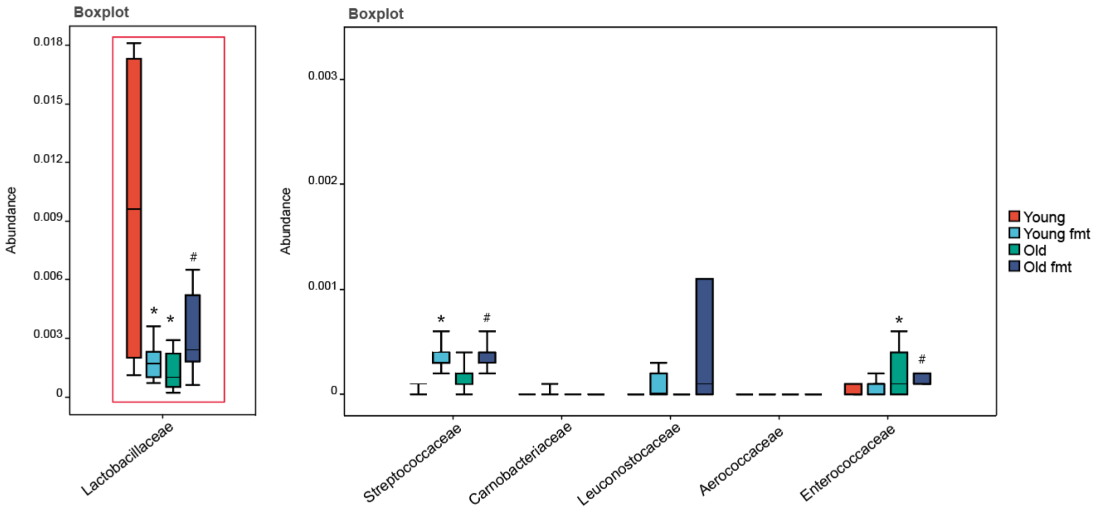

Supplementary Figure S3. Abundance of gut microbiota in *Bacilli-Lactobacillales*

**P*<0.05 compared with young control mice, #*P*<0.05 compared with aged mice.

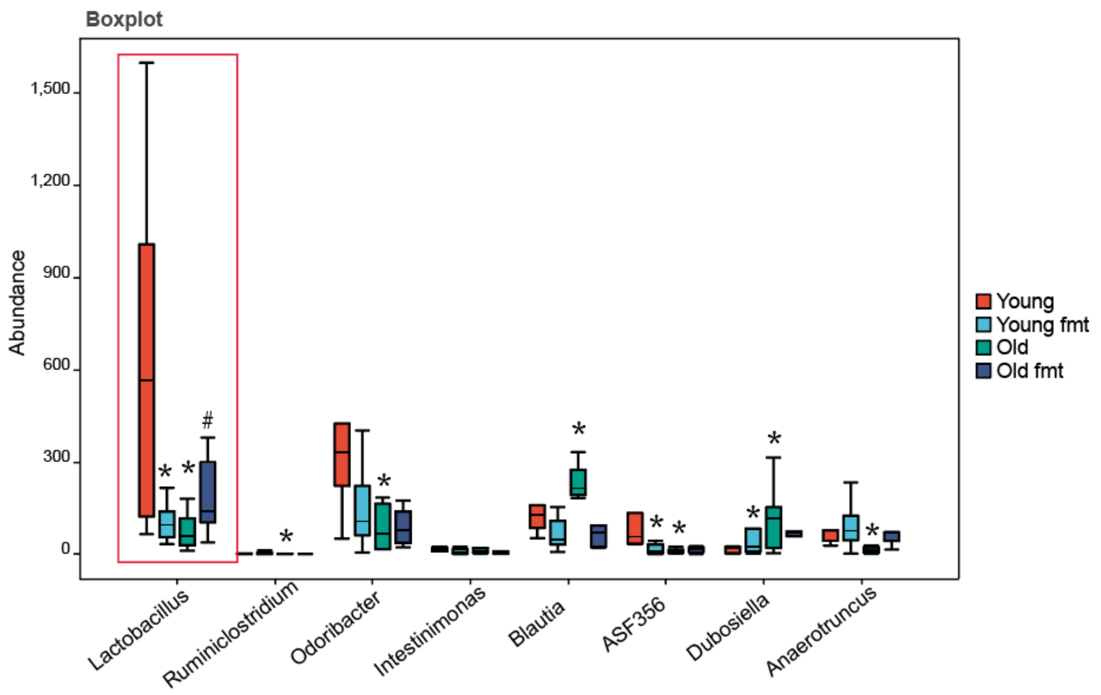

Supplementary Figure S4. Abundance of gut microbiota in genus rank *$P<0.05$ compared with young control mice, #$P<0.05$ compared with aged mice.

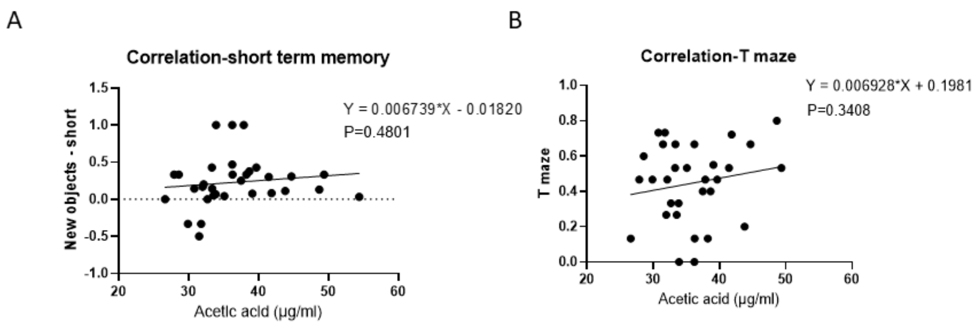

Supplementary Figure S5. Serum acetic acid levels do not correlate with mouse performance scores in short-term memory (A) or T-maze test (B)

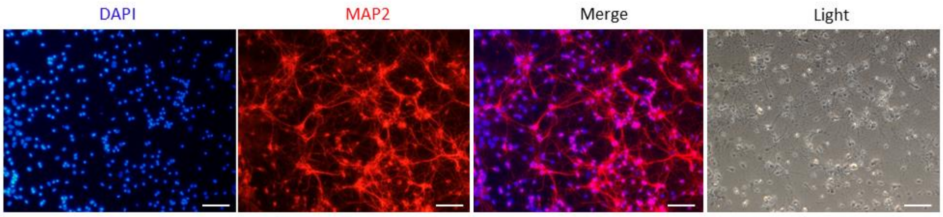

Supplementary Figure S6. Immunofluorescence signals of MAP2 in primary neurons of mouse hippocampus Blue for DAPI, red for MAP2. Magnification, 200×.

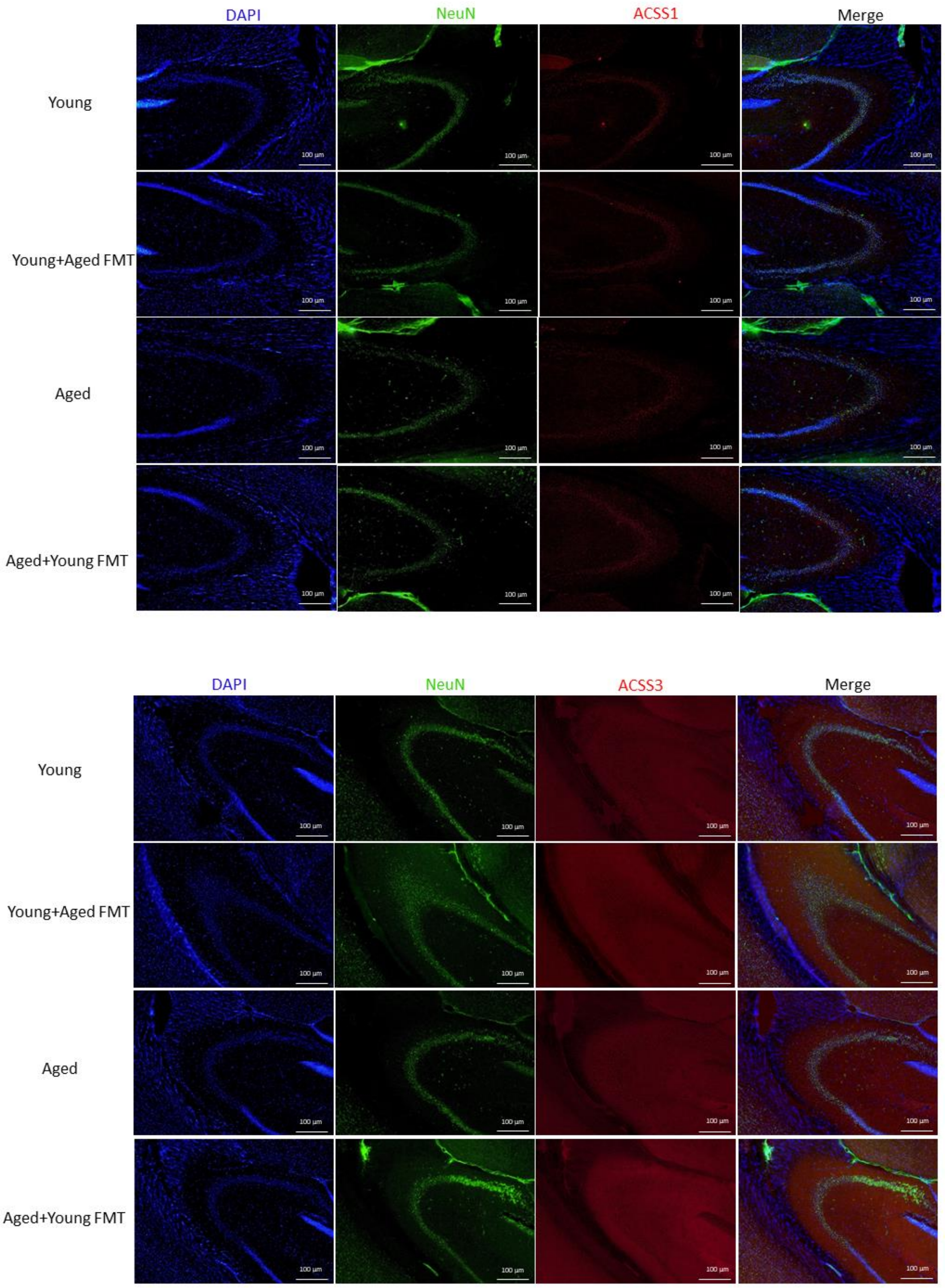

Supplementary Figure S7. Protein presence of ACSS1 and ACSS3 in CA3 area of mouse hippocampus
